# Thalassemia and Priapism: A Literature Review of a Rare Association

**DOI:** 10.7759/cureus.14335

**Published:** 2021-04-07

**Authors:** Sundus Sardar, Elrazi A Ali, Mohamed A Yassin

**Affiliations:** 1 Internal Medicine, Hamad Medical Corporation, Doha, QAT; 2 Department of Hematology, National Centre for Cancer Care and Research, Hamad Medical Corporation, Doha, QAT

**Keywords:** thalassemia, priapism, hematology

## Abstract

Thalassemia is a hematologic disorder caused by genetic mutation resulting in impaired hemoglobin chain production. Patients with thalassemia commonly experience complications such as anemia, blood transfusion-related issues, hepatic or cardiac involvement, and psychosocial impacts. Rarely, priapism has been associated with thalassemia as an initial presentation or subsequently occurring at any time in the disease course. Our literature review summarizes the reported cases of thalassemia-associated priapism and delves into underlying mechanisms of its pathophysiology and appropriate management.

## Introduction and background

Thalassemia is a hematologic disorder caused by mutations in the genes coding for hemoglobin chains. The most common causes of genetic disorders in humans are mutations within the β-globin gene, of which 350 β-thalassemia mutations have been identified to date [1]. Clinical manifestations of thalassemia are highly variable, ranging from asymptomatic in individuals with mild/silent mutations to mild hypochromic anemia, while other individuals may have life-long transfusion-dependent moderate to severe anemia and multi-organ involvement [2]. 

Individuals with thalassemia may experience complications, including but not limited to liver fibrosis and cirrhosis, cardiac failure, arrhythmias, and endocrinopathies [3]. Endocrine complications of thalassemia major commonly involve growth hormone and insulin-like growth factor (IGF-1) axis resulting in IGF-1 deficiency associated with growth hormone deficiency [4]. Adrenal abnormalities in patients with thalassemia were appropriately identified with low-dose adrenocorticotropic hormone (ACTH) test rather than standard dose ACTH, deeming it necessary to perform a low-dose ACTH test to identify possible adrenal insufficiency or underlying latent hypocortisolism, especially in thalassemia patients undergoing major surgical procedures [5,6]. 

Thalassemia patients may rarely present with or subsequently develop priapism, which is defined as a persistent penile erection lasting longer than four hours. Priapism can be classified into ischemic (veno-occlusive low flow), non-ischemic (arterial high flow), and stuttering (recurrent episodes) [7]. In most cases, the underlying etiology remains unidentified (primary/idiopathy). Secondary causes of priapism consist of intracavernous injections (iatrogenic); medications; hematological disorders; neurological involvement (spinal shock); trauma to the perineal, penile, or pelvic regions; infection (malaria, spider toxins); and metabolic disorders [7]. Priapism is rarely reported in patients with thalassemia. Clitoral priapism has been reported in other diseases; however, there are no cases reported in association with thalassemia. The underlying pathophysiology is not clear; however, the mechanism for the development of priapism is attributed to deranged autoregulation of penile circulation due to the nitric oxide pathway and phosphodiesterase enzyme activity [7]. 

## Review

**Figure 1:**
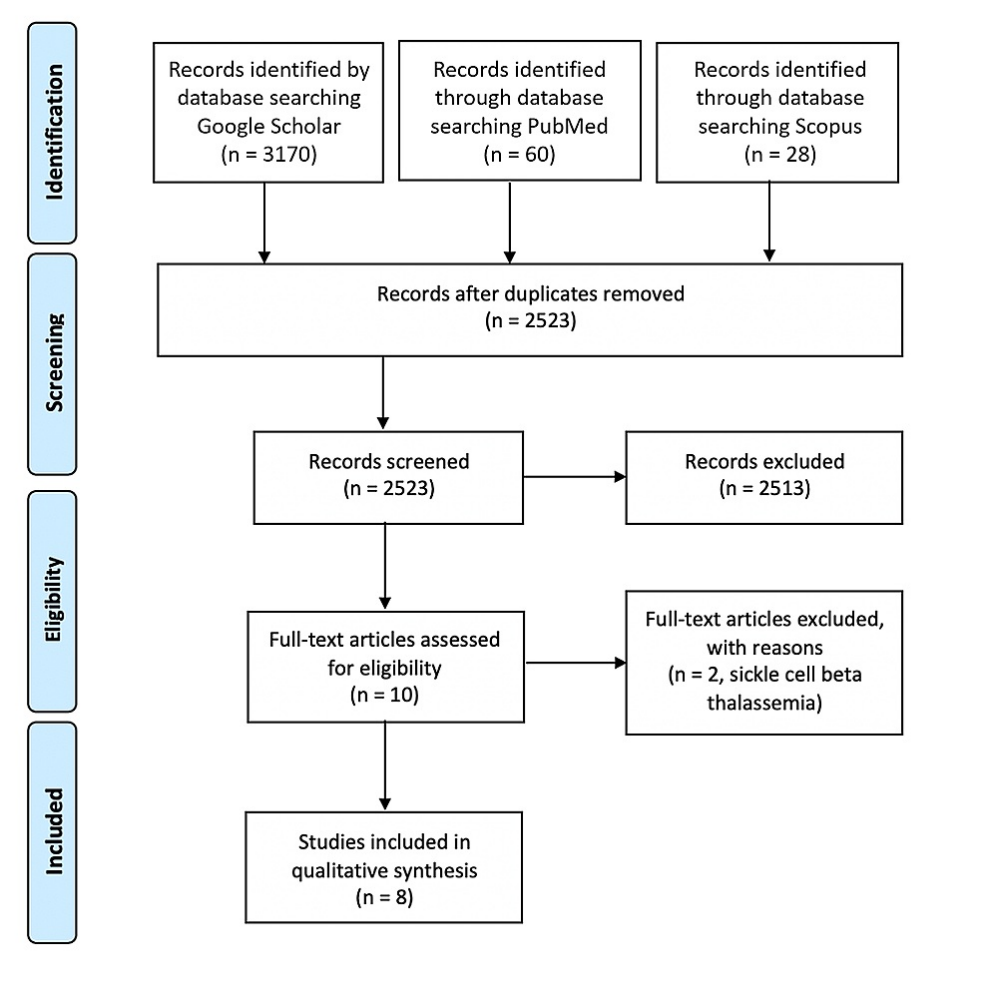
The PRISMA flow diagram illustrating the cases of thalassemia associated with priapism as initial presentation or subsequent development during disease course. Abbreviation: PRISMA, Preferred Reporting Items for Systematic Reviews and Meta-Analyses.

We conducted an extensive review of case reports, case series, and observational studies on thalassemia from 1986 to 2020 on PubMed, Scopus, and Google Scholar search engines to determine priapism's incidence as an initial presentation or subsequently occurring during the thalassemia disease course. All articles documenting priapism associated with thalassemia were included. Any cases of priapism associated with sickle cell beta-thalassemia or related to other diseases or medications were excluded (Figure 1). 

Eight appropriate articles (total nine cases) of priapism associated with thalassemia were identified [8-15]. Table 1 elicits thalassemia subtype, hematological parameters, management of priapism, and clinical outcome. Cases are arranged chronologically. Age groups reported range from 15 to 42 years. 

**Table 1:** Reported cases of priapism associated with thalassemia, arranged by chronological order WBC, white blood cell; Hb, hemoglobin; HbE, hemoglobin E; NA, not applicable; HU, hydroxyurea; DFX, deferasirox; IV, intravenous; BMT, bone marrow transplant.

Patient number	Author and year of publication [citation]	Age (years)	Time from onset of priapism to presentation and diagnosis	Type of priapism	Platelet count (x10^9^/L)	WBC (x10^9^/L)	Hb (g/dL)	Type of thalassemia	Intervention/Medication	Splenomegaly/Hepatomegaly	Outcome
1	Jackson, 1986 [8]	15	10-12h, 4 episodes post-splenectomy	NA	370	NA	8.5	Intermedia	Hydroxyurea Aspiration	Splenomegaly on initial presentation.	Four recurrent episodes, followed by complete resolution.
2	Macchia, 1990 [9]	NA	NA	NA	NA	NA	NA	Intermedia	NA	History of splenectomy	NA
3	Mankan, 2005[10]	42	24 hours	NA	274	6.9	10.5	Thalassemia minor	Aspiration, intracavernous phenylephrine injection	NA	Complete resolution
4	Sharma, 2008 [11]	32	4 hours prior to current presentation [suffered 20–25 self-remitting similar episodes in last 1 month]	NA	Normal [value not stated].	Normal [value not stated].	10.6	HbE beta-thalassemia [r IVS1-5[G-C]/b E mutation]	Puncture of corpus cavernosa	Hepatomegaly, history of splenectomy	Complete resolution.
5	Ziaee, 2008 [12]	15	7 hours	Ischemic	156.7	15.4	10	Major beta-thalassemia	Oxygenation, hydration, epinephrine injections thrice.	History of splenectomy	Complete resolution
6	Tzortzis, 2009 [13]	19	At age of 13 years, then recurrent episodes 6 weeks post-splenectomy	Ischemic stuttering	NA	NA	NA	Thalassemia intermedia	Aspiration, irrigation, self-injections with phenylephrine, oral antiplatelet agent, sildenafil citrate as prevention strategy	Hypersplenism on initial presentation.	Nonpainful recurrent episodes with decreased frequency and not requiring medical management.
7	Mallat, 2014 [14]	30	1 month	Stuttering priapism	NA	NA	9.2	Beta thalassemia intermedia	Cloridrate propanolol 3 drops daily, HU, DFX, cholecalciferol, folic acid	NA	Complete resolution
8		35	1 month		NA	NA	7		Puncture of corpus cavernosa	Hepatomegaly	Complete resolution
9	Öz, 2017 [15]	17	Second day of hospitalization	Ischemic, vaso-occlusive	NA	18.3	8.2	Thalassemia major	IV hydration, alkalization, oxygen therapy, allogeneic BMT, deferosirox as chelation therapy, busulfan, clophosphamide with Mesna	NA	Complete resolution

Thalassemia and its associated complications adversely impact the quality of life of affected individuals, including adverse effects on their overall health, school performance, mental health status, and the physical, social and psychological aspects of their lives [16,17]. Additionally, thalassemic patients with priapism as a complication or presenting concern experience further worsening of quality of life due to impairment of sexual function and the possible risk of penile fibrosis and, subsequently, permanent erectile dysfunction. Thus far, there are no reports of cases with thalassemia-associated clitoral priapism.

With advancements in thalassemia management and identifying factors impacting the quality of life, many patients have good outcomes and are successfully married [18]. In adolescents and young male patients affected with chronic disease (e.g., hemoglobinopathies, failure of pubertal growth, absence or delay of sexual development), infertility and sexual dysfunction are well-established disturbances secondary to hypogonadism and impaired spermatogenesis [19]. Infertility negatively impacts these individuals' future quality of life and is also a predictor of stress in any existing or forthcoming relationships. In these patients, the impact of priapism on quality of life, sexual function, and physical wellness can be evaluated and quantified using the Priapism Impact Profile questionnaire [20].

While priapism is rarely seen in thalassemic patients, a postulated mechanism proposes a cellular mechanism owing to increased blood viscosity as demonstrated by thrombocytosis or elevated nucleated red blood count (NRBC) [21]. Moreover, intravascular stasis may cause thrombotic occlusion of efferent venules in the corpora cavernosa, resulting in intracorporal sludging, fibrosis, and impairment of the erectile mechanism. Another proposed functional mechanism involves altering the molecular determinants of erectile response due to abnormal nitric oxide activity in the penile tissue, downregulation of phosphodiesterase-5 activity, and the overresponse of the penile smooth muscle. In the hemolytic state of thalassemia, nitric oxide hemostasis is impaired due to nitric oxide consumption, resulting in vasoconstriction [21].

Priapism is a rare entity in the spectrum of thromboembolic disease. Splenectomized thalassemic patients are predisposed to developing a thromboembolic event (TEE) due to elevated NRBC, thrombocytosis, and platelet aggregation. Moreover, factors associated with earlier development of TEE post-splenectomy include transfusion naivety, thrombocytosis, and elevated NRBC due to the thrombogenic potential of negatively charged membranes [22].

As evidenced by our data, most thalassemia and priapism patients had low hemoglobin values (mean hemoglobin level was 9.14 ± 1.32 g/dL). Thus far, in the literature, 55.5% (five out of nine cases) of thalassemic patients with priapism were diagnosed as thalassemia intermedia. All cases of priapism reported in thalassemic patients had complete resolution. A higher prevalence of priapism is evident in post-splenectomized thalassemic patients [8,9,11,13].

The management of priapism in thalassemic patients includes conservative management similar to that in individuals with sickle cell disease, oxygenation, hydration, and analgesics. Further treatment consists of a supertransfusion regimen, erythropoiesis, irrigation, and intracavernosal alpha-agonist injections with phenylephrine every five minutes after aspiration of 10 mL to 20 mL of blood [23]. For cases of priapism refractory to conservative treatment modalities, the next therapeutic step involves surgical intervention and spongiocavernous shunt insertion [24].

Long-term complications of priapism in thalassemic individuals include penile fibrosis and erectile dysfunction, with discernable negative impacts on quality of life due to impaired sexual function and troublesome reproductive issues. A recent case reported treatment of erectile dysfunction in thalassemic men with the administration of transurethral E1-prostaglandins such as alprostadil as an effective, non-invasive therapy [25].

## Conclusions

Priapism is a rare presentation in patients with thalassemia with a significant impact on these individuals' quality of life. While only a few cases have been reported, priapism is documented as a rare association more commonly seen in thalassemia intermedia and post-splenectomized thalassemic individuals. Early identification and appropriate management of priapism in thalassemic patients are essential to prevent longstanding erectile dysfunction.
